# Atovaquone Suppresses Triple-Negative Breast Tumor Growth by Reducing Immune-Suppressive Cells

**DOI:** 10.3390/ijms22105150

**Published:** 2021-05-13

**Authors:** Nehal Gupta, Shreyas Gaikwad, Itishree Kaushik, Stephen E. Wright, Maciej M. Markiewski, Sanjay K. Srivastava

**Affiliations:** 1 Department of Biomedical Sciences, Texas Tech University Health Sciences Center, Amarillo, TX 79106, USA; nehal.gupta@ttuhsc.edu (N.G.); stephen.wright@ttuhsc.edu (S.E.W.); 2 Department of Immunotherapeutics and Biotechnology, Center for Tumor Immunology, and Targeted Cancer Therapy, Texas Tech University Health Sciences Center, Abilene, TX 79601, USA; Shreyas.gaikwad@ttuhsc.edu (S.G.); i.kaushik@ttuhsc.edu (I.K.); maciej.markiewski@ttuhsc.edu (M.M.M.); 3 Department of Internal Medicine, Texas Tech University Health Sciences Center, Amarillo, TX 79106, USA

**Keywords:** myeloid-derived tumor-suppressor cells, atovaquone, repurposing, triple-negative breast cancer, regulatory T cells, cytokines, C5aR1, HIF1α, RPS19

## Abstract

A major contributing factor in triple-negative breast cancer progression is its ability to evade immune surveillance. One mechanism for this immunosuppression is through ribosomal protein S19 (RPS19), which facilitates myeloid-derived suppressor cells (MDSCs) recruitment in tumors, which generate cytokines TGF-β and IL-10 and induce regulatory T cells (Tregs), all of which are immunosuppressive and enhance tumor progression. Hence, enhancing the immune system in breast tumors could be a strategy for anticancer therapeutics. The present study evaluated the immune response of atovaquone, an antiprotozoal drug, in three independent breast-tumor models. Our results demonstrated that oral administration of atovaquone reduced HCC1806, CI66 and 4T1 paclitaxel-resistant (4T1-PR) breast-tumor growth by 45%, 70% and 42%, respectively. MDSCs, TGF-β, IL-10 and Tregs of blood and tumors were analyzed from all of these in vivo models. Our results demonstrated that atovaquone treatment in mice bearing HCC1806 tumors reduced MDSCs from tumor and blood by 70% and 30%, respectively. We also observed a 25% reduction in tumor MDSCs in atovaquone-treated mice bearing CI66 and 4T1-PR tumors. In addition, a decrease in TGF-β and IL-10 in tumor lysates was observed in atovaquone-treated mice with a reduction in tumor Tregs. Moreover, a significant reduction in the expression of RPS19 was found in tumors treated with atovaquone.

## 1. Introduction

Based on the presence or absence of estrogen receptors, progesterone receptors and human epidermal growth receptor 2 (HER2), breast cancer is mainly classified into four types: luminal A, luminal B, HER2+ and triple-negative breast cancer [1]. There are several treatment options for breast cancer based on this classification. However, this classification system does not take into consideration the role of the immune system, which has been shown to contribute significantly to cancer regression.

Triple-negative breast cancer (TNBC) is characterized by the negative expression of estrogen and progesterone receptors and HER2 [2]. TNBC has unique biological characteristics and is considered to be more aggressive than any other type of breast cancer, as there are no approved target therapies [3]. Cytotoxic chemotherapy is suggested for the treatment of TNBC. Interestingly, studies have shown that some chemotherapeutics affect the immune system to kill cancer cells [4]. However, a recent study has indicated immune evasion as one of the mechanisms of resistance to chemotherapy in TNBC [5]. Moreover, TNBC is shown to be immunogenic, therefore modulating the immune system to eradicate the disease could be helpful [6,7]. In addition, several studies have shown that immunotherapy drugs are effective for treatment of breast cancer [8]. The role of immune system in cancer growth and elimination has been increasing. However, there is a paucity of information examining the impact of current drugs on the immune system. In the current study, we have evaluated the effect of atovaquone on the immune system in suppressing breast tumor growth.

Myeloid-derived suppressor cells (MDSCs) consist of a heterogeneous population of myeloid cells that includes myeloid progenitors, granulocytes, macrophages, dendritic cells as well as immature mononuclear cells [9]. MDSCs are known to suppress the immune system and thus play a crucial role in promoting tumor growth and metastasis [10,11,12]. Studies indicated that the efficacy of immunotherapy is reduced with the presence of MDSCs [13]. Another type of immunosuppressive cells are regulatory T (Treg) cells, which also promote cancer development and progression [14,15]. Several studies have shown that targeting Tregs could be helpful for breast-cancer treatment and preventing metastasis [16,17].

Transforming growth factor β (TGF-β) is a ubiquitous cytokine and is involved in normal cell proliferation, differentiation and homeostasis [18,19]. Interleukin-10 (IL-10) is an anti-inflammatory cytokine that is produced by wide variety of cells. These cytokines have dual effects on tumor growth [20,21,22,23]. Both TGF-β and IL-10 function at the crossroads of immune stimulation and immune suppression in cancer and their role in immune pathology is unclear [21,24]. It has been reported that production of IL-10 and TGF-β by MDSCs is crucial for the induction of Tregs [25,26]. In addition, IL-10 plays a vital role in tumor metastasis and facilitates tumor cell proliferation [27]. Several studies have found that TGF-β and IL-10 levels are significantly higher in breast-cancer patients [28,29]. TGF-β is also angiogenic in nature, which helps in tumor cell survival. TGF-β antagonists were shown to be effective in preventing metastasis of lung and breast cancer [30,31]. Therefore, TGF-β could be a potential target for cancer therapy [32,33].

Ribosomal protein S19 (RPS19), another molecule associated with immunosuppression, is the endogenous ligand for complement component 5a receptor 1 (C5aR1) [34]. RPS19 interacts with C5aR1 expressed on tumor infiltrating MDSCs. This interaction facilitates MDSCs recruitment in tumors leading to tumor growth [35,36]. RPS19 is known to be associated with a high incidence of cancer in humans. Increased risk of cervical cancer was found to be associated with single-nucleotide polymorphisms in the *RPS19* gene [37]. In addition, RPS19 mRNA expression was found to be upregulated in colon carcinoma [38]. Its expression was also found to be upregulated in several breast and ovarian cancer cell lines [39]. A recent study has shown novel immunosuppressive effects of RPS19. RPS19 promotes generation of Tregs with the induction of immunosuppressive cytokine TGF-β [39]. Therefore, reducing its level in tumor cells should impede the development of tumors by decreasing RPS19 mediated immunosuppression.

Another crucial factor within the tumor microenvironment which is responsible for immune suppression and resistance is hypoxia. Hypoxia is one of the hallmarks of the tumor microenvironment and hypoxia-inducible factors (HIF) are the main molecular transcriptional mediators in hypoxia response [40,41]. Ample evidence suggested that intratumoral hypoxia and HIF1α play an essential role in the regulation of tumor immune responses [42,43]. HIF1α promotes Treg cells recruitment to the tumor sites and also promotes immune suppressive activity of MDSCs and tumor-associated macrophages (TAM) [44]. Therefore, therapeutic strategies targeting HIF1α could be beneficial for antitumor immune responses.

Atovaquone is an anti-protozoal drug used for the treatment of Pneumocystis pneumonia and malaria. A few studies, including studies from our lab, have suggested that atovaquone possesses strong anticancer activity [45,46,47,48,49]. A recent study from our lab has shown that atovaquone significantly suppresses the growth of several breast-cancer cells in vitro and in vivo by inhibiting HER2/β-catenin signaling [49]. To understand the detailed mechanism of action, we evaluated the effects of atovaquone on immune cells associated with breast tumor growth suppression.

In the current study, effect of atovaquone was evaluated on MDSCs and Tregs in different breast tumor models. Our results showed that atovaquone significantly inhibited breast tumor growth. In addition, atovaquone suppressed MDSCs and Tregs in blood and tumor cells with a reduction in immunosuppressive cytokines TGF-β, IL-10 and RPS19. This is the first report on the immunomodulatory effects of atovaquone.

## 2. Results

### 2.1. Atovaquone Inhibits Breast Tumor Growth 

**Figure 1:**
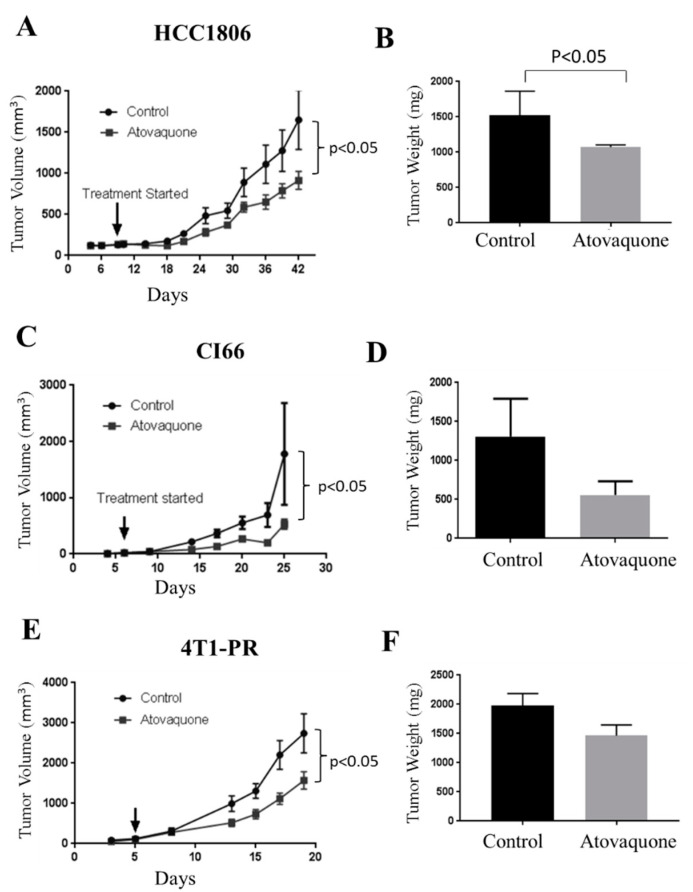
Atovaquone suppresses breast tumor growth. Human peripheral blood mononuclear cells (8 × 10^6^) cells were injected intraperitoneally 7 days after HCC1806 tumor cells’ implantation in NCG/SCID mice; *n* = 5/group. (**A**) HCC1806 tumor growth curves. (**B**) Endpoint tumor weights. (**C**) Tumor growth curve of CI66 cells; *n* = 6/group. (**D**) Bar graph representing average tumor volume of control and atovaquone-treated mice at day 25; 4T1 paclitaxel-resistant cells were implanted in right and left flanks of female Balb/c mice, and treatment was 25 mg/kg atovaquone daily; *n* = 10/group. (**E**) Tumor growth curve. (**F**) Tumor weight from control and atovaquone-treated group at day 20. Values were plotted as mean ± SEM. Statistically significant at *p* < 0.05 when compared with control.

To determine the efficacy of atovaquone in inhibiting the growth of breast tumor in vivo three independent experiments were performed. In the first experiment, 2 × 10^6^ cells HCC1806 human breast-cancer cells were implanted orthotopically in right 3rd mammary fat pad of NCG/SCID mice (*n* = 5). About 8 × 10^6^ PBMCs were injected intraperitoneally after 7 days of tumor implantation. We injected human PBMCs in mice after implantation of tumors to allow better interaction of PBMCs with the tumor cells. In addition, this also allowed maturation of PBMC cells into MDSCs with a human phenotype. Atovaquone (50 mg/kg) was administered by oral gavage every day once the average tumor volume reached about 100 mm^3^. The experiment was terminated on day 42 due to excessive tumor burden in control mice. After 42 days, atovaquone significantly retarded mammary tumor growth by 45% (Figure 1A). The weight of tumors collected from the atovaquone-treated group was approximately 30% less than the control group (Figure 1B). Moreover, we did not observe any significant change in the weight of the mice throughout the study, indicating no toxicity of atovaquone at this dose (Supplementary Materials Figure S1).

In another experiment, 0.1 × 10^6^ murine CI66 breast-cancer cells were orthotopically implanted in the left mammary fat pad of female Balb/c mice (*n* = 6). Once each mouse attained about 70–100 mm^3^ tumors, mice were randomly divided into two groups. Control mice received vehicle only whereas the treatment group of mice received 50 mg/kg atovaquone every day by oral gavage. A similar study was performed by orthotopic implantation of 4T1 paclitaxel-resistant tumors (4T1-PR) in female Balb/c mice (*n* = 10). Resistance to paclitaxel was developed by us and the cells were about >100 fold resistant to paclitaxel [50]. Our results showed 70% reduction in the size of CI66 tumors and 42% reduction in 4T1-PR tumors (Figure 1C,E). At the end of the experiment, mice were euthanized, tumors were dissected and weighed. We observed a 30% reduction in tumor weight in the atovaquone-treated mice as compared to the control mice. These results suggest strong inhibitory effects of atovaquone on human and mice breast-tumor growth (Figure 1D,F).

### 2.2. Reduction in Myeloid-Derived Suppressor Cells (MDSCs) with Atovaquone Treatment

**Figure 2:**
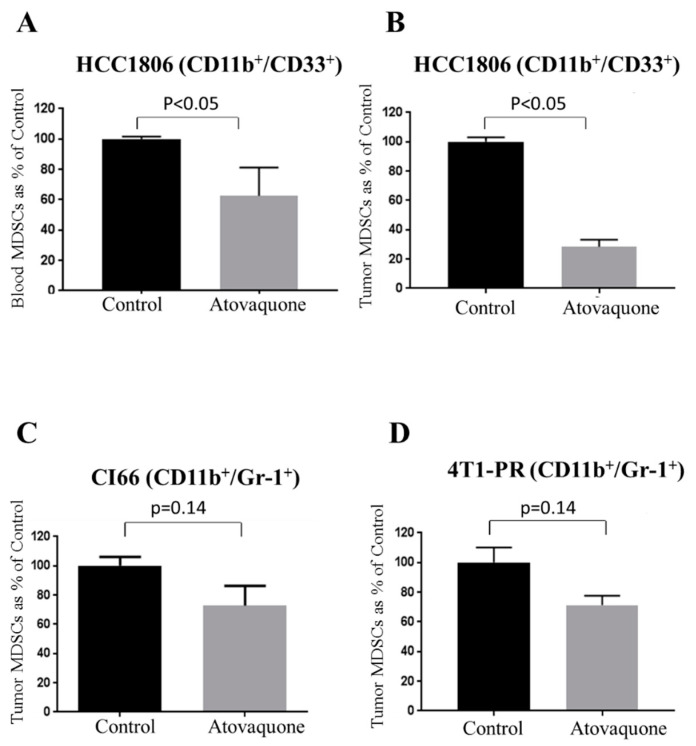
Effect of Atovaquone on MDSCs. Peripheral blood mononuclear cells (PBMCs) were collected from the blood obtained from HCC1806 tumor-bearing mice. (**A**) Bar chart representing percent MDSCs which were stained with CD11b and CD33 human antibodies; *n* = 4. Tumors from control and atovaquone-treated group in all three in vivo experiments were isolated, processed and suspended into single-cell suspension with the help of gentle MACS dissociator using tumor dissociator kit. Percentage of MDSCs from (**B**) HCC1806 tumor cells (stained with CD11b/CD33 human antibodies); *n* = 3 (**C**) CI66 tumors (stained with CD11b/Gr-1 mouse antibodies); *n* = 3 and (**D**) 4T1-PR tumors (stained with CD11b/Gr-1 mouse antibodies); *n* = 3. Data shown as mean ± SEM. A *p*-value less than 0.05 was considered to be significant.

Next, we evaluated the effect of atovaquone on immunosuppressive MDSCs collected from different in vivo experiments described above as well as in method section using specific markers. MDSCs are known to promote the growth of tumors [51]. High infiltrations of MDSCs are also known to increase stem cell like characteristics in tumors [52]. PBMCs were collected from the blood of control and atovaquone-treated mice and double stained with CD11b and CD33 antibodies against MDSCs. Our results showed that atovaquone treatment resulted in 40% reduction in the blood MDSCs (Figure 2A). Further, single-cell suspension from control and atovaquone-treated tumors were double stained with either CD11b/CD33 (human) or CD11b/Gr-1 (mouse) [53]. Our results demonstrated that atovaquone treatment suppressed 70% CD11b^+^CD33^+^ MDSCs obtained from HCC1806 tumors (Figure 2B). We also observed about 25 and 30% reduction in tumor MDSCs collected from CI66 and 4T1-PR tumors, respectively (Figure 2C,D). These observations indicated that atovaquone treatment suppressed MDSCs, which play a significant role in promoting tumor growth.

### 2.3. Reduction of Regulatory T Cells (Tregs) by Atovaquone Treatment

**Figure 3:**
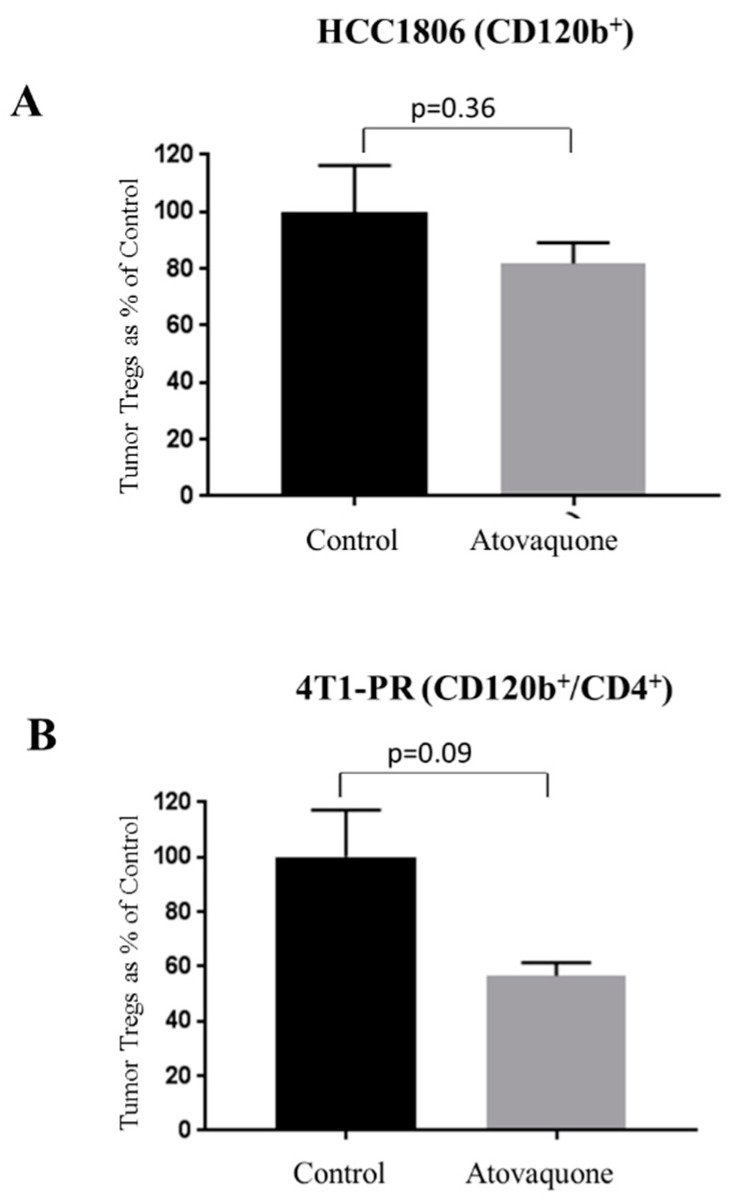
Atovaquone suppresses regulatory T cells (Treg) in HCC1806 and 4T1-PR tumors. Tumors were removed from mice bearing HCC1806 and 4T1-PR tumors at the end of the experiment. Tumors from control and atovaquone-treated groups were processed and suspended into single-cell suspension, using a tumor dissociator kit. Modulation of Treg cells was monitored by immunostaining with CD120b/CD4 and analyzed by flow cytometry. (**A**) Percent Tregs from control and atovaquone-treated groups stained with CD120b in HCC1806 tumor cells. (**B**) %Tregs double stained with CD120b and CD4 mouse specific antibodies in 4T1PR tumor cells. Values were plotted as mean ± SEM from *n* = 4 samples.

Another type of immunosuppressive cells are Tregs. MDSCs plays a critical role in the induction of Tregs via IL-10 [25]. Hence, it was important to evaluate the effect of atovaquone on Treg cell population. We used CD120b and CD4 markers to label Tregs. HCC1806 tumors collected from control and atovaquone-treated groups were dissociated into single-cell suspension and stained with CD120b. Our results indicated that atovaquone treatment showed slight a modest 12% reduction in Tregs from HCC1806. In another experiment, single-cell-suspension 4T1-PR tumors from control and atovaquone-treated mice were double stained with CD120b and CD4 antibodies against Tregs. We observed about 50% reduction in Tregs collected from 4T1-PR tumors with atovaquone treatment, indicating an increase in immune surveillance in mouse bearing human tumors (Figure 3A,B).

### 2.4. Atovaquone Suppressed Tumor Cytokines

**Figure 4:**
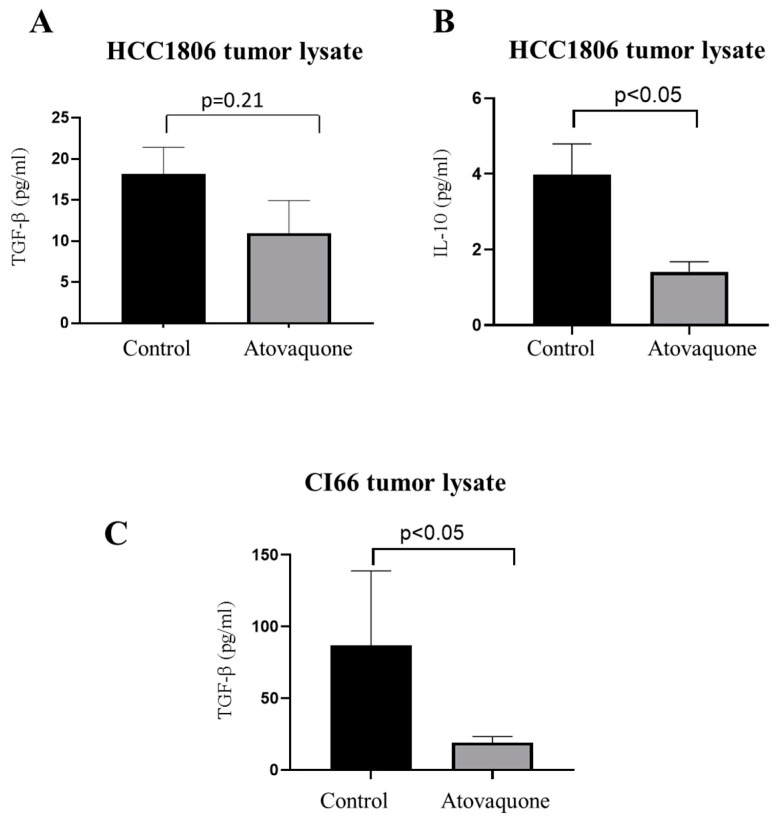
Reduction of immune-suppressive cytokines with atovaquone treatment. Equal amount of protein from HCC1806, as well as CI66 tumors from control and atovaquone-treated mice, was used to perform ELISA assay for TGF-β and IL-10. Bar graph representing (**A**) TGF-β (HCC1806 tumor lysate), (**B**) IL-10 (HCC1806 tumor lysate) and (**C**) TGF-β (CI66 tumor lysate) levels from control and treatment groups. Data shown as mean ± SEM from at least three individual tumor samples. Statistically significant at *p* < 0.05 when compared with control.

Next, we wanted to determine the mechanism responsible for atovaquone suppressing Tregs and MDSCs. For this purpose, orthotopically implanted HCC1806 tumors from immunocompromised NCG/SCID mice were examined for immunosuppressive cytokines after termination of the experiment. TGF-β and IL-10 are potent immunosuppressive cytokines which induce Tregs that in turn allow cancer cells to escape immune surveillance. This mechanism largely contributes to tumor progression [25]. ELISA assay was performed to estimate TGF-β and IL-10 levels from the tumors obtained from control and atovaquone-treated mice. Our results demonstrated that atovaquone treatment resulted in 13% and 40% reduction of TGF-β and IL-10 levels, respectively, indicating a reduction of immunosuppression in the tumor microenvironment (Figure 4A,B).

To confirm these observations, we also performed ELISA assay for TGF-β in tumor lysate of CI66 tumors and observed 60% reduction in TGF-β in atovaquone-treated tumors as compared to control tumors (Figure 4C). These results indicate reduction of immunosuppressive cytokines in the tumor microenvironment after atovaquone treatment.

### 2.5. Atovaquone Reduces the Expression of the Immunosuppressive Ribosomal Protein S19 (RPS19) and Induces Apoptosis

**Figure 5:**
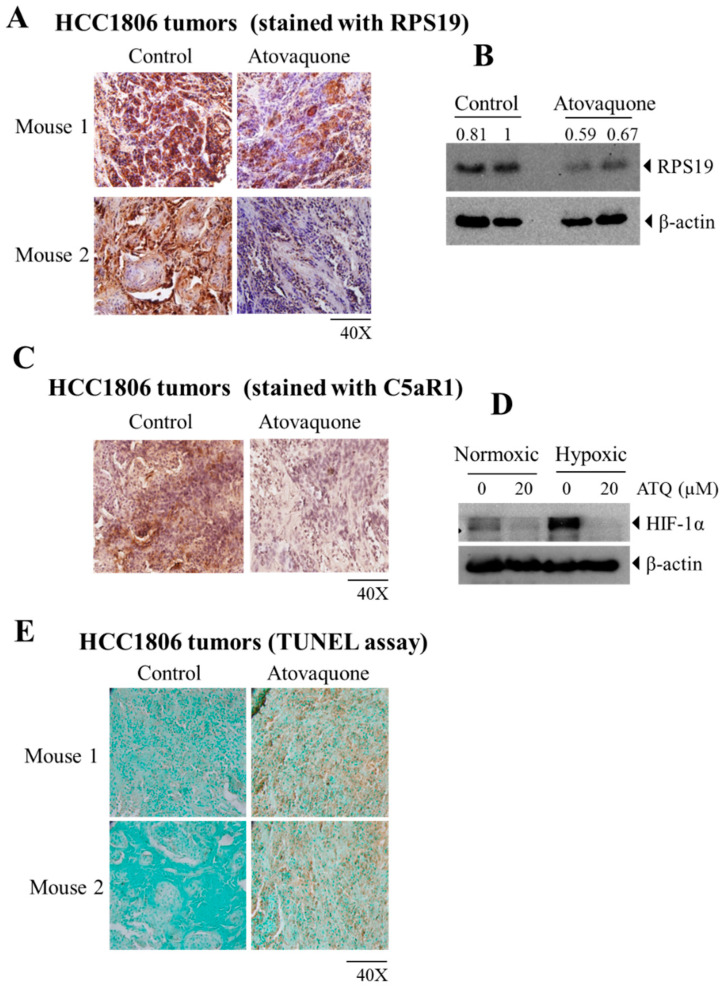
Reduction in the expression of RPS19 with increased apoptosis after atovaquone treatment. Tumors were isolated from mice bearing HCC1806 tumors at the day of termination. A part of tumor was fixed in formalin for immunohistochemical analysis, as well as for Western blot analysis. (**A**) Treated and untreated HCC1806 tumor sections were analyzed for RPS19 by IHC. (**B**) Western blot analyses showing the expression of RPS19 in control and treated HCC1806 tumors. (**C**) Expression of C5aR1 in atovaquone-treated and untreated HCC1806 tumors. (**D**) HIF-1α expression in HCC1806 cells treated with or without atovaquone (ATQ) under both normoxic and hypoxic conditions by Western blotting. (**E**) Representative images of excised tumor sections from control and atovaquone-treated group analyzed by TUNEL assay. Each section indicates a tumor from an individual mouse.

RPS19 promotes generation of regulatory T cells while reducing infiltration of CD8 + T cells into tumors [39]. Reducing RPS19 in tumor cells or blocking the interaction between MDSCs’s C5a receptor and RPS19 decreases RPS19-mediated immunosuppression, which leads to impairment in breast tumor growth [39]. At the end of the experiment, HCC1806 tumors were collected and analyzed by IHC and Western blotting. We observed a significant reduction in the expression of RPS19 in tumors treated with atovaquone as analyzed by IHC (Figure 5A). Quantification of stained samples from IHC showed 75% reduction in RPS19 staining in the atovaquone-treated group compared to control. In line with the IHC results, Western blot analysis also showed a reduction in the expression of RPS19 with atovaquone treatment in HCC1806 tumor lysates (Figure 5B). In addition, atovaquone treatment suppressed the expression of C5aR1 in human breast tumors HCC1806 as shown by IHC analysis (Figure 5C). This suggests that the immunomodulatory effects of atovaquone in breast cancer could also be due to a reduction in the expression of RPS19.

Hypoxia and HIF1α have been known for a long time as an intricate part of the tumor microenvironment. Studies have also shown that hypoxic stress promotes immunosuppressive pathways involving MDSCS, Tregs and macrophages, and thus promotes tumor progression [43]. Thus, we determined the expression of the immune suppressive marker, RPS19 under hypoxic condition and found that RPS19 levels were slightly higher in hypoxic condition (Supplementary Materials Figure S2). Therefore, we wanted to check if atovaquone can reduce HIF1α levels. For this purpose, we treated the human breast-cancer cell line HCC1806 with 20 µM atovaquone under normoxic and hypoxic conditions. Our results showed a reduction in the expression of HIF1α with atovaquone treatment under both normoxic, as well as hypoxic conditions, suggesting a significant role of atovaquone in suppressing hypoxia in breast-cancer cells (Figure 5D).

Furthermore, we analyzed the effects of atovaquone on apoptosis. TUNEL (terminal deoxynucleotidyl transferase dUTP nick end labeling) assay is a method to identify apoptosis by detecting DNA fragmentation. When analyzed by TUNEL assay, our results indicated increased apoptosis in tumors obtained from the atovaquone-treated group as compared to the control (Figure 5E). This suggests that atovaquone treatment may have a direct effect on tumor cells. This is consistent with our previous studies where we have shown that atovaquone treatment reduced 4T1 and CI66 tumors in mice [49].

## 3. Discussion

TNBC is one of the deadliest cancers. Resistance to chemotherapy leads to recurrence of breast cancer after conventional therapy. Recent advancements have shown the effectiveness of immunotherapy to treat TNBC [54,55,56]. Immunotherapy utilizes patient’s own immune system to recognize and kill cancer cells. However, cancer cells induce various immunosuppressive cells, such MDSCs, Tregs, NK cells and dendritic cells, and therefore, they evade the immune system. MDSCs and Tregs suppress immune potentiating cells and are increased during cancer [57]. A previous study from our lab has shown that the antitumor activity of atovaquone was associated with inhibition of HER2/β-catenin signaling [49]. Our current study evaluated the immune modulation by atovaquone, correlating with its antitumor effects.

A few studies have shown the potent anticancer effects of atovaquone in various cancer models [45,46,47,48]. We evaluated the antitumor efficacy of atovaquone in breast tumors. We observed 45% tumor suppression in HCC1806 after 42 days of 50 mg/kg every day atovaquone treatment. On the other hand, we observed approximately 70% suppression of CI66 tumors when mice were treated with 50 mg/kg atovaquone daily for 25 days. Paclitaxel is an FDA approved drug for breast-cancer treatment. However, patients get resistant to paclitaxel hindering chemotherapy. In vivo efficacy of atovaquone was further evaluated in paclitaxel-resistant tumor model. Treatment with 25 mg/kg atovaquone resulted in a reduction in tumor volume of 4T1 paclitaxel-resistant tumors by 42%. The difference in the efficacy of atovaquone could be attributed to the difference in the cell line type as HCC1806 is the human breast-cancer cell line, whereas CI66 cells originated from murine breast tumors. The 4T1 paclitaxel-resistant cells were developed in our lab by gradually exposing the 4T1 cells to paclitaxel [50].

Atovaquone acts as a strong antitumor agent by inhibiting mitochondrial respiration and by inhibiting survival pathways such as signal transducer and activator of transcription 3 (STAT3) and human epidermal growth receptor 2 (HER2) [45,47]. Previous studies have shown that inhibition of STAT3 and HER2 reduces MDSCs and Treg activity and thus inhibits cancer progression [58,59,60]. The dose of atovaquone used in the current study in mice was 50 mg/kg per day, which is about 4-fold less than its upper anti-malarial dose used clinically by humans. The human equivalent dose (HED) after conversion comes out to be 4.1 mg/kg per day or 250 mg per day for females weighing 60 kg.

Wesolowski et al. discussed various ways to inhibit MDSCs for treatment of cancer [61]. Hence, we decided to evaluate the effect of atovaquone on MDSCs. MDSCs consist of immature myeloid cells that are upregulated in cancer. MDSCs are known for their potent immunosuppressive activity and increased tumor progression [62]. Intriguingly, MDSCs accumulation highly depends on the tumor model used [63]. To mimic the human conditions, we injected PBMCs isolated from human blood after 7 days of HCC1806-breast-tumor implantation in NCG/SCID mice. We examined the effect of atovaquone on human MDSCs collected from blood, as well as tumors, using the surface marker CD11b/CD33. In addition, we also evaluated the effect of atovaquone on MDSCs in murine tumor xenografts, using the surface markers CD11b/Gr-1. Our results showed a significant reduction in MDSCs with atovaquone treatment in both human and murine tumors.

MDSCs also facilitate the production of Tregs, which, in turn, downregulate cell-mediated immunity [64]. Our results showed a reduced population of CD120b/CD4 positively stained cells obtained from atovaquone-treated tumors as compared to control groups. One mechanism by which MDSCs induce Treg activation is by the production of IL-10 and TGF-β [26,64]. We observed a reduction in TGF-β and IL-10 levels in atovaquone-treated tumors, when analyzed by ELISA assay. Reduction in the expression of a novel immunosuppressive molecule, RPS19 and its interacting receptor C5aR1 was also observed in atovaquone-treated tumors. In addition, atovaquone-induced apoptosis in HCC1806 tumors, as shown by TUNEL assay. Moreover, hypoxia and HIF1α are considered as an integral components of tumor microenvironment. Interestingly, atovaquone treatment suppressed the expression of HIF1α under both normoxic and hypoxic conditions.

**Figure 6:**
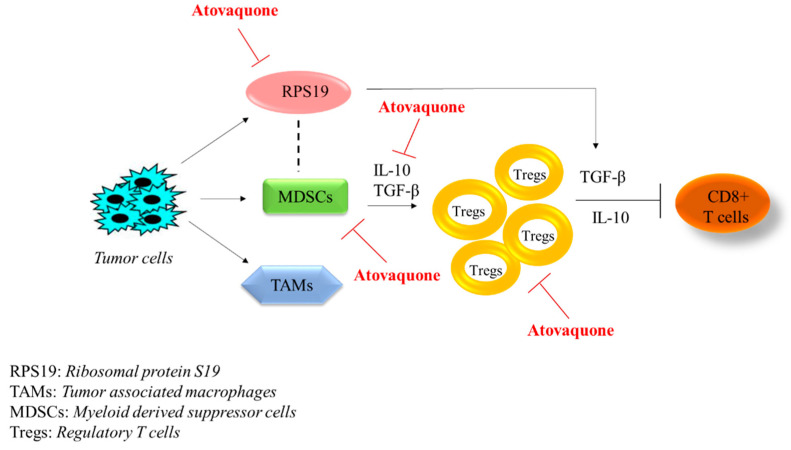
Schematic diagram depicting the role of atovaquone in immune system.

Taken together, our study reports a novel antitumor mechanism of atovaquone (Figure 6).

Tumors secrete various factors which promote the accumulation of heterogeneous population of TAMs, MDSCs and immunosuppressive protein RPS19 in the tumor microenvironment. MDSCs induce Tregs’ activation by the production of IL-10 and TGF-β. Furthermore, Tregs suppress CD8+ cytotoxic T cells’ function through secretion of IL-10 or TGF-β from Tregs and tumors. The dotted line in Figure 6 indicates the interaction between RPS19 and MDSCs in tumor microenvironment that induces the production of immunosuppressive cytokines, including TGF-β. Atovaquone (ATQ) reduces the expression of RPS19, MDSCs, Tregs and immunosuppressive cytokines IL-10 and TGF-β in tumor cells.

Our results indicate that atovaquone reduces the expression of RPS19 in tumors, thus reducing MDSCs and Tregs with the reduction in immunosuppressive cytokines, TGF-β and IL-10. Previous studies from our lab and others have shown the antitumor effects of atovaquone [45,46,48]. It appears that the mechanism of immune suppression of atovaquone by MDSCs in our model may be through TGF-β and IL-10, which are potent immunosuppressive cytokines and induce Tregs that allow cancer cells to escape from immune surveillance. This study is of high interest because MDSCs are one of the major components of the tumor microenvironment with potent immunosuppressive activity and atovaquone has the potential to suppress MDSCs. Our study provides impetus to further explore the immunotherapeutic effects of atovaquone.

## 4. Materials and Methods

### 4.1. Cell Culture

Two murine mammary adenocarcinoma cell lines CI66 and 4T1 and one human squamous breast carcinoma cell line HCC1806 were used in this study. CI66 cells were kindly provided by Dr. Rakesh Singh (UNMC, Omaha, NE, USA) [65,66]. A kind gift of HCC1806 cells by Dr. Sophia Ran (Southern Illinois University-School of Medicine, Springfield, IL, USA) is greatly appreciated. These cell lines were maintained in DMEM supplemented with 10% FBS and 1% PSN (Penicillin, Streptomycin and Neomycin). Moreover, 4T1 paclitaxel-resistant cells (4T1-PR) were developed by us by gradually exposing the 4T1 cells to paclitaxel for several months and were cultured in DMEM supplemented with 10% FBS with 50 nM paclitaxel [50]. All the cell lines were periodically authenticated by short tandem repeats (STR) analysis in the core facility of Texas Tech University, Lubbock (Texas). All the cell lines used for performing experiments were below passage number 20.

### 4.2. Reagents and Chemicals

All the antibodies CD11b-FITC (#130-098-085), CD33-PE (#130-098-896), Gr-1-PE (#130-102-426), CD120b (TNF-R II)-PE (#130-104-697), CD4-FITC (#130-102-779) and blocking reagent (#130-059-901) were purchased from Miltenyl biotech. Atovaquone (#A7986) was purchased from sigma. Atovaquone suspension (Mepron) was purchased from Prasco laboratories for in vivo studies. RPS19 antibody (#ab155994) and actin antibody (Sigma; #A5441) were obtained from Abcam and Sigma, respectively.

### 4.3. Human PBMCs Isolation from Buffy Coat

Human blood was obtained from the Oklahoma Blood Institute, from anonymous donors. The buffy coat was diluted 2-fold with sterile PBS. Then 10 mL of Ficoll-Paque reagent (GE Lifesciences) was added to a 50 mL tube over which 25 mL of diluted buffy coat was gently layered. The tubes were further centrifuged at 400× *g*, for 40 min, at 20 °C, without applying any brakes. Following this step, a clear layer of PBMCs was observed at the interface of Ficoll-Paque and plasma, which was collected and washed with sterile PBS.

### 4.4. Tumor Therapy Model

All animal experiments were carried out according to approved Institutional Animal Care and Use Committee protocols at the Texas Tech University Health Sciences Center (Amarillo, TX, USA). Tumor volume was measured by using a Vernier caliper, as described previously [67]. For the first in vivo study, female NCG/SCID mice (4–6 weeks old) were obtained from Charles River. Exponentially growing HCC1806 cells were harvested, trypsinized and washed with 1XPBS. Then 100μL of cell suspension comprising 2 × 10^6^ cells in 1:1 mixture of PBS and Matrigel was injected orthotopically into the right 3rd mammary fat pad of 10 recipient mice. Once the tumor was palpable (70–80 mm^3)^ at day 7 of the tumor cells’ implantation, 8 × 10^6^ human PBMCs were injected in all mice intraperitoneally. The next day, after PBMCs injection, mice were randomly divided into two groups, with 5 mice in each group (*n* = 5). The treatment group received 50 mg/kg atovaquone by oral gavage every day, till day 42.

In another tumor model, 0.1 × 10^6^ CI66 cells in a mixture of PBS and matrigel (1:1) were injected in 4th mammary fat pad of 12 female Balb/c mice. The average weight of the mice at the day of tumor cells injection was 16 g. Mice were randomly divided into two groups, with 6 mice in each group (*n* = 6), once tumors reached approximately 80 mm^3^. The treatment group mice were treated with 50 mg/kg atovaquone by oral gavage every day, until day 25. At day 22, two mice from the control group were having difficulty moving due to the tumor burden; thus, they were sacrificed. At day 24, another mouse from the control group and two mice from the treatment group were found dead due to the tumor burden. Remaining mice from the both the groups were euthanized at day 25.

The third in vivo study, using 4T1-PR cells, was described by us previously [50]. Briefly, 0.07 × 10^6^ 4T1-PR cells in 1:1 PBS/matrigel were implanted in right and left 3rd mammary fat pad of 10 female Balb/c mice. When tumor volume reached 70–100 mm^3^ (day 5), mice were randomly divided into two groups (*n* = 10). Mice were treated with 25 mg/kg atovaquone by oral gavage every day, till day 19.

Tumors from all the in vivo experiments from the control and treated groups were removed aseptically, weighed and processed for further analysis. Tumors were dissociated into single suspension, using a tumor dissociation kit (Miltenyl Biotech, Bergisch Gladbach, Germany), with the help of a gentleMACS dissociator (Miltenyl Biotech), as per manufacturer’s instructions. Moreover, a part of tumor was kept for Western blot and immunohistochemical analysis.

### 4.5. PBMC Collection from Mouse Blood and Fluorescence Cytometric Analysis

At the end of the experiment, PBMCs were collected from the blood of treated and control mice to analyze the effects of atovaquone on PBMCs. Blood was collected from mice by cardiac puncture and diluted 10-folds, using RBC lysis buffer, and incubated at room temperature on a shaker for 20 min. The tubes were further centrifuged at 300× *g* for 10 min at 4 °C. This process was repeated 2 times. The cells were then washed with FACS buffer (2% heat inactivated fetal bovine serum and 2 mM ethylenediaminetetraacetic acid (EDTA) in PBS) after one wash with PBS. The collected PBMCs were blocked with FcR blocking reagent at 4 °C in dark for 15 min. Then, the cells were washed with FACS buffer by adding 1 mL of FACS buffer and centrifuging at 300 × *g*for 10 min. Then appropriate antibodies (prepared in FACS buffer) were added to cells and the samples were incubated one ice in dark for 30 min. The samples were then re-suspended in 300 μL of FACS buffer after washing with PBS and analyzed by using a flow cytometer (Accuri C6 flow cytometer, BD Biosciences, San Jose, CA, USA), as described by us previously [68,69]. The monocyte and lymphocyte cell populations were used to determine MDSCs and Tregs respectively in control and atovaquone-treated groups. Mean channel intensities were used to analyze the data.

### 4.6. Enzyme-Linked Immune Sorbent Assay

ELISA assay was performed for TGF-β and IL-10, using and ELISA kit from Affymetrix, Ebioscience, according to the manufacturer’s instructions. HCC1806 tumors were collected from control and atovaquone-treated groups of NCG/SCID mice at the end of the experiment (day 42). Tumors were lysed by using RIPA buffer, and protein was estimated by using Bradford reagent (Bio-Rad, Hercules, CA, USA). Equal amounts of protein (60–70 μg) from control and atovaquone-treated tumor lysate samples were used to perform ELISA assay. Similarly, ELISA assay was also performed in CI66 tumors from the control and atovaquone-treated groups for TGF-β.

### 4.7. Immunostaining for Tissue Sections

The immunohistochemistry (IHC) was performed as described by us previously [70]. Tumors were aseptically removed and fixed with 10% formalin for 24 h, at room temperature. On the next day, the formalin solution was replaced with 70% ethanol until further use. Tumors were then paraffinized through a series of solvents, using tissue processor. Paraffin-embedded tissues were sectioned into approximately 10 µm–thick sections using microtome (Leica Microsystems Inc., Buffalo Grove, IL, USA). After deparaffinization and rehydration, tissue sections were boiled in sodium citrate buffer (pH 6.0) for antigens retrieval and were further incubated in 3% hydrogen peroxide methanol solution. The sections were then washed and blocked with 5% goat serum (Life Technologies, Carlsbad, CA, USA #16210064) for 1 h, at room temperature. Subsequently, the tissue sections were incubated with primary antibodies, overnight, at 4 °C, for RPS19 (#ab181365, 1:200) and C5aR1 (#sc-53797, 1:200). On the next day, primary antibody was removed, and the sections were washed with wash buffer, followed by 30 min of incubation with Ultravision ONE HRP polymer (Thermo Fisher Scientific, Waltham, MA, USA), as per the manufacturer’s instructions. Subsequently, sections were washed with wash buffer and incubated with DAB Plus chromogen for 15–20 min. The sections were counterstained with hematoxylin and dehydrated. The slides were mounted by using Permount (Thermo Fisher Scientific, Rockland, IL, USA) and analyzed under a bright field Olympus microscope (Olympus America Inc., Brooklyn Park, MN, USA).

TUNEL assay was carried out by using a FragEL DNA Fragmentation Detection Kit (Calbiochem, San Diego, CA, USA) according to manufacturer’s protocol.

### 4.8. Hypoxia Treatment and Western Blot Analysis

For hypoxia treatment, cells were treated with 20 µM of atovaquone and then exposed to hypoxic conditions (1% O_2_) for 72 h. After 72 h, cells were collected for Western blot analysis. Firstly, cells were washed with 1× PBS and lysed with 4% (*w*/*v*) CHAPS in urea-Tris buffer on ice. The cell lysate was then cleared by centrifugation at 14,000 g for 30 min. Bradford reagent was used to quantify the amount of protein in the lysate. Cell lysate containing 25–40 μg protein was resolved by 10% sodium dodecyl sulfate–polyacrylamide gel electrophoresis (SDS–PAGE), and the proteins were transferred onto polyvinylidene fluoride (PVDF) membrane. The PVDF membrane was then blocked with 5% non-fat dry milk in Tris-buffered saline, for 1 h, at room temperature. The membranes were probed with primary antibodies, overnight, at 4 °C, against HIF-1α (Cell Signaling Technology, Danvers, MA, USA; #3716, rabbit mAb, 1:1000 dilution), RPS19 ((#ab181365, 1:1000 dilution) and β-actin (Sigma, St. Louis, MO, USA; #A5441, mouse mAb, 1:2000 dilution). Subsequently, the membranes were incubated with either anti-mouse (Sigma; #SAB4600224) or anti-rabbit (Perkin Elmer, Waltham, MA, USA; #NEF812001EA) secondary antibody (1:2000) horseradish peroxidase conjugate, for 2 h, at room temperature, and detected by chemiluminescence kit (ThermoScientific, Waltham, MA, USA; #32106). Films were scanned and blots were quantified by using UN-SCAN-IT gel 7.1 software. The basal level of RPS19 was initially evaluated in all the cell lines (Supplementary Materials Figure S3).

Tumor lysates were prepared by homogenizing the control and atovaquone-treated HCC1806 tumors in PBS, lysed using RIPA buffer and subjected to Western blot analysis, as described above. The primary antibody used for RPS19 was purchased from Abcam (Cambridge, UK; #ab181365, 1:1000 dilution).

### 4.9. Statistical Analysis

Statistical analysis was performed by using Prism 7.0 (GraphPad software Inc., San Diego, CA, USA). Results were represented as means ± SD or SEM with minimum value of *n* = 3. Data were analyzed by Student’s *t*-test or Fischer (F) test. A *p*-value less than 0.05 was considered statistically significant.

Abbreviations: MDSC, myeloid-derived suppressor cell; Treg, T regulatory cells; PBMC, peripheral blood mononuclear cell; RPS19, ribosomal protein S19; C5aR1, complement component 5a receptor 1; HIF1α, hypoxia inducible factor 1 α.

## Data Availability

Not applicable.

## Supplementary Materials

The following are available online at https://www.mdpi.com/article/10.3390/ijms22105150/s1, Supplementary Figure S1: Weight of the mice bearing HCC1806 tumors throughout the study. Supplementary Figure S2: RPS19 and HIF1α expression in HCC1806 cells under hypoxic condition by Western blot analysis. Supplementary Figure S3: Basal level of RPS19 in HCC1806, CI66 and 4T1-PR cells by Western blot analysis.
