# Supplementary figures and images for: Atovaquone Suppresses Triple-Negative Breast Tumor Growth by Reducing Immune-Suppressive Cells
Source: Int J Mol Sci. 2021 May 13;22(10):5150. doi: 10.3390/ijms22105150 (PMC8152242; doi:10.3390/ijms22105150)

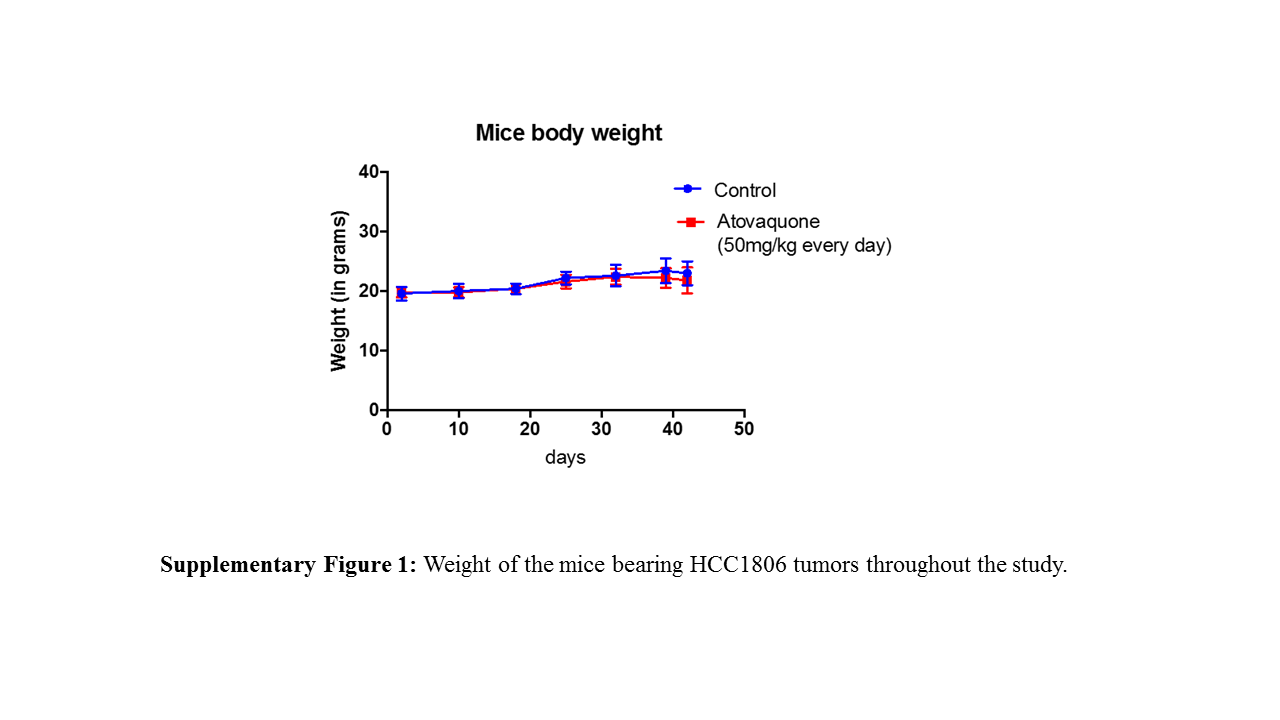

Supplement: Supplementary file 1 [file ijms-22-05150-s001.zip › Fig S1.TIF]

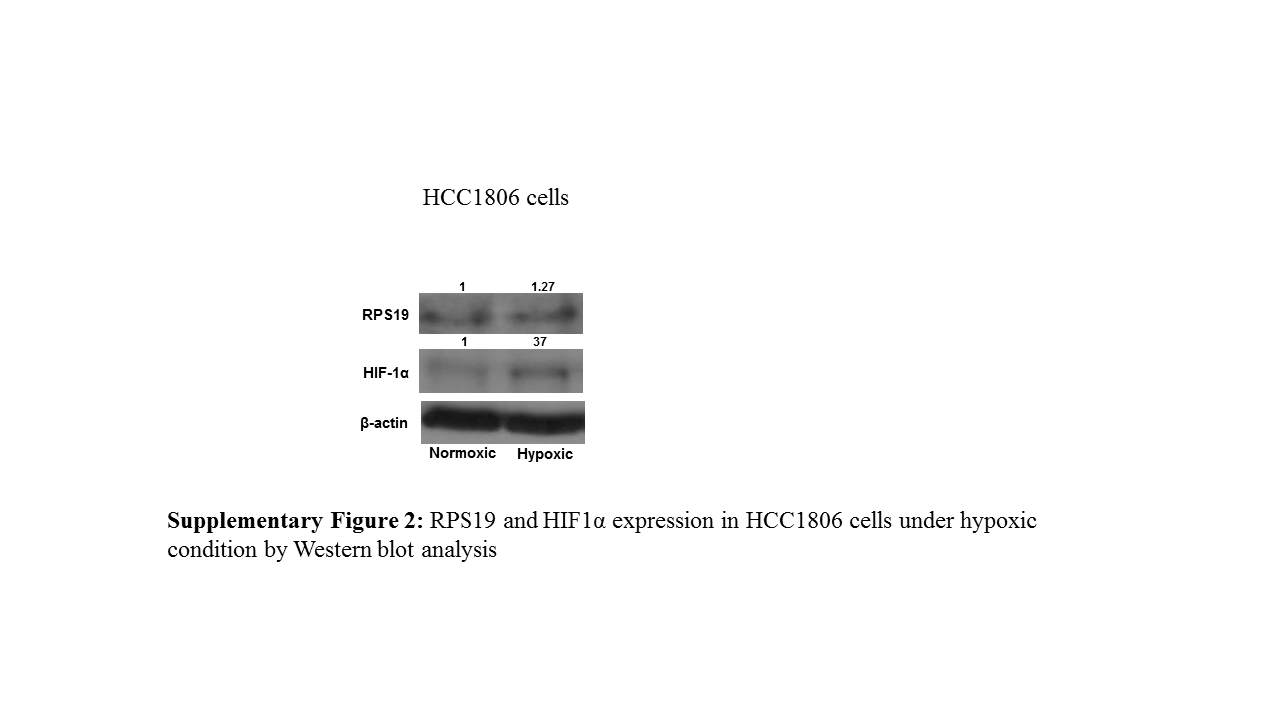

Supplement: Supplementary file 1 [file ijms-22-05150-s001.zip › Fig S2.TIF]

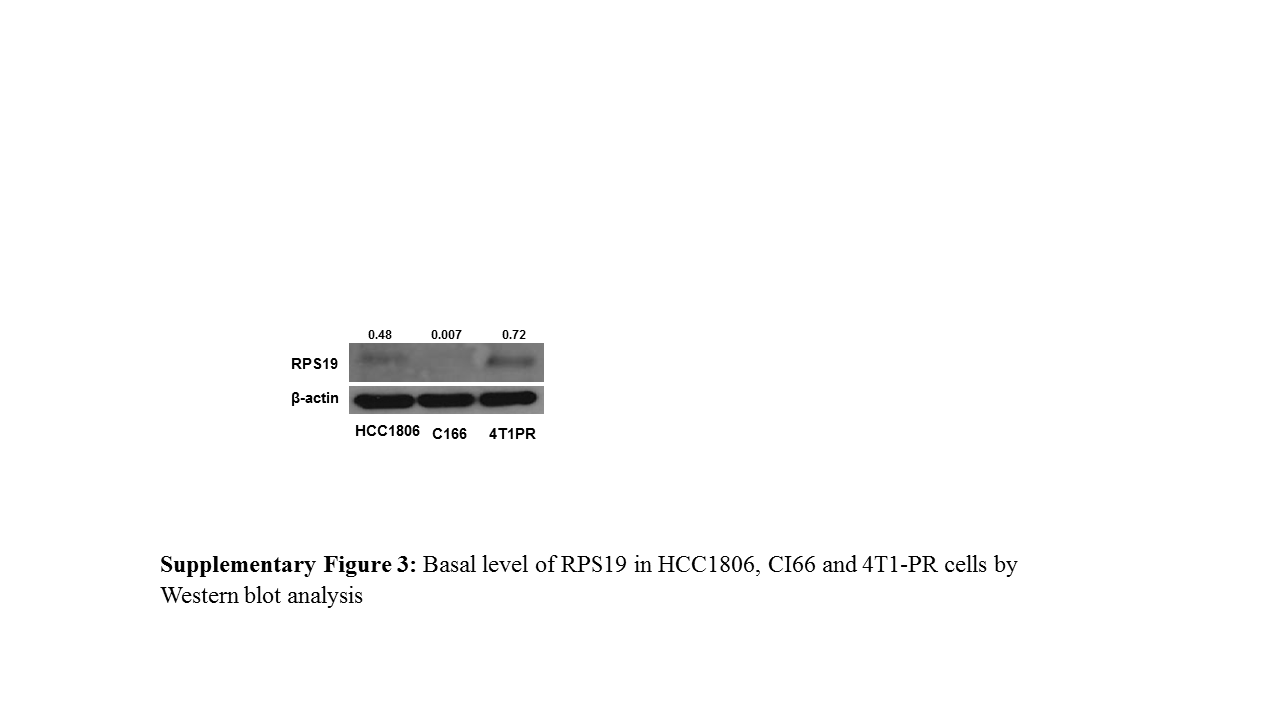

Supplement: Supplementary file 1 [file ijms-22-05150-s001.zip › Fig S3.TIF]
